# The downed and dead wood inventory of forests in the United States

**DOI:** 10.1038/sdata.2018.303

**Published:** 2019-01-08

**Authors:** Christopher W. Woodall, Vicente J. Monleon, Shawn Fraver, Matthew B. Russell, Mark H. Hatfield, John L. Campbell, Grant M. Domke

**Affiliations:** 1 USDA Forest Service, Northern Research Station, Durham, NH, 03824, USA; 2 USDA Forest Service, Pacific Northwest Research Station, Corvallis, OR, 97331, USA; 3 University of Maine, Orono, ME, 04469, USA; 4 University of Minnesota, St Paul, MN, 55108, USA; 5 USDA Forest Service, Northern Research Station, St. Paul, MN, 55108, USA

**Keywords:** Forest ecology, Forestry, Plant ecology, Fire ecology

## Abstract

The quantity and condition of downed dead wood (DDW) is emerging as a major factor governing forest ecosystem processes such as carbon cycling, fire behavior, and tree regeneration. Despite this, systematic inventories of DDW are sparse if not absent across major forest biomes. The Forest Inventory and Analysis program of the United States (US) Forest Service has conducted an annual DDW inventory on all coterminous US forest land since 2002 (~1 plot per 38,850 ha), with a sample intensification occurring since 2012 (~1 plot per 19,425 ha). The data are organized according to DDW components and by sampling information which can all be linked to a multitude of auxiliary information in the national database. As the sampling of DDW is conducted using field efficient line-intersect approaches, several assumptions are adopted during population estimation that serve to identify critical knowledge gaps. The plot- and population-level DDW datasets and estimates provide the first insights into an understudied but critical ecosystem component of temperate forests of North America with global application.

## Background & Summary

Downed dead wood (DDW) can be defined as detrital components of forest ecosystems, including fallen twigs and small branches (fine woody debris) and fallen tree stems and large branches (coarse woody debris). Downed dead wood plays a central role in many forest ecosystem functions^1,2^, including wildlife habitat^3^, biodiversity of deadwood-dependent organisms^4^, tree regeneration^5,6^, wildfire risk and behavior^7,8^, nutrient cycles^9^, and carbon (C) stocks and cycling^10–12^. Downed dead wood data are used to inform National Greenhouse Gas Inventories^11,13,14^, and when coupled with live tree inventory data^15,16^, they provide a robust quantification of forest changes over time^17^. Finally, as global change is projected to increase the frequency and intensity of natural disturbances that cause tree mortality and hence create DDW (e.g., invasive pests, droughts, windstorms, wildfires), we can expect expanded interest and use of DDW datasets to address wildfire risks, forest sustainability, and C estimation and modelling.

The inventory and associated monitoring of DDW began with the advent of modern forest fire fighting in the 1960’s where rapid field assessments of forest fuels (e.g., logging residue^18^) were essential. These inventories were often not systematic across any nation, but rather tactical in nature or limited to specific forests. With the adoption of comprehensive national forest monitoring initiatives in the 1990’s (e.g., Santiago Declaration^19^) and the greenhouse gas reporting requirements for the land sector in the United Nations Framework Convention on Climate Change ([UNFCCC] i.e., dead wood C pool of the forest land category^20^), the first national-scale DDW inventories were initiated (e.g., Sweden^21^). More recently, several developed nations such as Sweden, Mexico, United States (US), and Australia^12,22^ have completed at least one systematic national DDW inventory. These countries employ various sampling techniques and intensities but with the common goal of filling a major knowledge gap in global forest monitoring.

**Figure 1:**
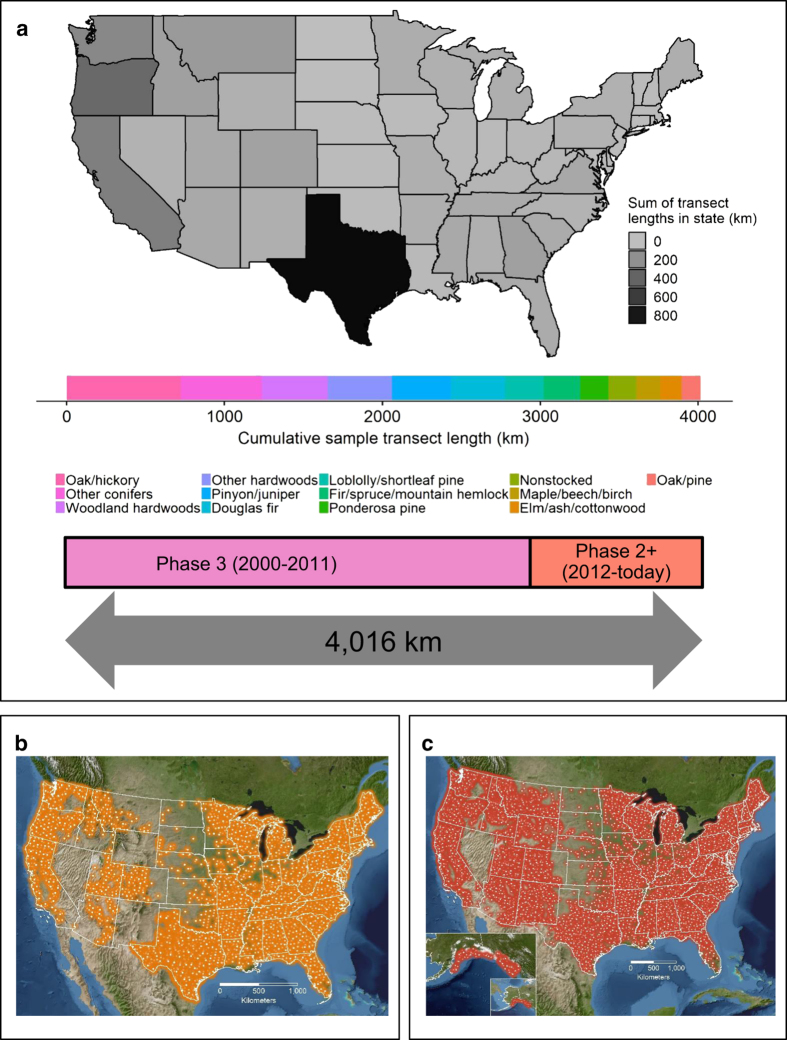
The plot network and transect information associated with the downed woody materials inventory of US forests. (**a**) The downed woody materials inventory has sampled a cumulative transect length of 4,016 km across 13 primary forest types, a length nearly equivalent to the distance between the most eastern and western points of the continental US (4,313 km). (**b**) Locations of inventory plots 2002–2010. (**c**) Locations of inventory plots 2012–2016. Plot locations are aligned with the distribution of forest across the United States.

The US Department of Agriculture Forest Service’s Forest Inventory and Analysis (FIA) program is the primary data source regarding the extent, condition, status, and trends of forest resources in the United States^23^. Although the FIA program has been in operation since the 1920’s, it had traditionally focused on estimating timber volumes on productive forest land. With the passage of the 1999 Farm Bill, the US Congress authorized the FIA program to expand its inventory program to all US forests (including non-timberlands such as wilderness areas or low productivity sites) and expand data collection to include forest ecosystem attributes beyond live trees: standing dead trees, understory vegetation, downed dead wood, soils, lichens, and tree crowns^24^. The FIA program began implementing a national inventory of DDW in 2001^11^ with data collected from 2002 onward currently publicly available (See Data Citations 1 and 2). FIA’s inventory serves as perhaps the largest publicly available DDW dataset across a continental scale, with plots in nearly every US state, and with cumulative sample transects spanning a distance nearly equivalent to the distance across the coterminous United States (Fig. 1). Describing these data, as well as the continuous monitoring program and recent modifications, is paramount to not only forest C science^25^, but also to emerging bioenergy efforts^26–28^.

## Methods

### Primer on downed dead wood sampling

As the inventory of DDW is a relatively new practice compared to that of live trees, various field methods are being used to measure and estimate DDW population parameters^12^. Most national inventories have adopted either fixed-area plots^29^ or line intersect sampling (LIS^30–32^) approaches with several other efficient sampling techniques available but not yet adopted at national scales^12^. Building upon its success with LIS for forest fuel assessments, while also considering the proven field efficiency of LIS, the US FIA adopted LIS for its national DDW inventory. Downed dead wood are only sampled in inventory plots, or portions of inventory plots, that lie in a forest land condition. Forest land is defined as having at least 10 percent canopy cover of live tree species or the potential to support such cover if recently cut/disturbed along with a spatial size requirement of ~0.4 ha and at least ~36.6 m in width. Line-intersect sampling itself consists of arraying one-dimensional sampling transects across forest domains of interest with any piece of DDW intersecting a transect being considered part of the sample. Population estimators are constructed based on the length of transect, diameter of the DDW piece, and the attributes of interest measured from each sampled piece. Understanding the LIS protocols and resulting data structure are essential for correct interpretation and use of the DDW data contained herein, because data records are organized according the LIS sampling strategy (e.g., transect *versus* piece records). As the US DDW inventory has improved since it was initiated, the sampling design and plot protocols have been modified to improve repeatability and efficiency, although they remain based on LIS.

Although the US DDW inventory provides a strategic-scale assessment of fundamental DDW population parameters such as volume and biomass, the evolving science of DDW dynamics continues to point to the complexity associated with DDW ecology that cannot be captured by the current inventory program. For example, the emerging role of microbial communities^33^ in governing DDW decay processes at regional scales suggests additional information is needed to fully accommodate the input of DDW estimates into soil biogeochemical models and associated Earth system modeling.

### Downed Dead Wood Definitions

As there have been revisions to both the DDW (alternatively referred to as Down Woody Materials in FIA program documentation and databases) population definitions and sample protocols from 2002 to the present, this data description will document components common to all field seasons (2002 to present) and exclude DDW components not remeasured such as shrub/herb cover. Components of the DDW population included in the national sampling effort are: coarse woody debris (sometimes assembled into piles), fine woody debris, duff, and litter. The duff and litter components are included in the DDW inventory to provide a more complete quantification of fuels for fire danger rating assessments.

Coarse woody debris (CWD) is defined as dead and downed pieces or portion of pieces of wood that meet the following criteria:

1. Diameter of at least 7.61 cm along a length of at least 0.15 m (0.91 m for inventories prior to 2012) for decay classes 1–4, and at least 12.7 cm above the duff layer along a length of at least 1.52 m for decay class 5 (for decay class definitions see FIA field guide^34^).
2. Detached from the bole of a standing live/dead tree. If still partially rooted, the lean angle must be more than 45 degrees from vertical. Standing dead trees with a lean angle less than 45 degrees from vertical are tallied as snags during other phases of the forest inventory.
3. Branched and forked pieces are considered as individual CWD pieces and tallied accordingly. For each piece, a main bole is determined as the fork with the largest diameter, with all branches and secondary forks considered separate CWD pieces that must meet minimum size specifications.
4. If CWD pieces become fractured, whether lengthwise or in broken sections, each portion is treated as a separate piece.

While most CWD pieces are tallied individually, occasionally there are accumulations so large that it is neither practical nor safe to attempt individual measurement of constituent, individual pieces of CWD using the standard LIS transect. This typically occurs as a result of harvest, but there may be large accumulations of wood along rivers, avalanche paths, or after large windthrow events. These distinct accumulations of CWD pieces, which we will refer to as piles, are measured using separate protocols.

Fine woody debris (FWD) is defined as down woody pieces with diameter less than 7.6 cm. FWD does not include dead branches attached to standing trees or shrubs, dead foliage, bark fragments, or small pieces of decomposed wood. The DDW component of FWD is further divided into 3 sizes classes that align with wildlife behavior modeling widely adopted in North America (e.g. US National Fire Danger Rating System^35^) based on transect diameter: small FWD (0.00–0.62 cm), medium FWD (0.63–2.54 cm), large FWD (2.55–7.60 cm).

Litter is defined as a forest-floor layer of freshly fallen leaves, needles, twigs, cones, bark chunks, dead moss, dead lichens, dead herbaceous stems, and flower parts^36^. Duff is defined as an organic forest-floor layer, just below litter, consisting of well-decomposed leaves and other organic material^36^. Individual plant parts should not be recognizable in the duff layer.

The definition of DDW populations of interest for any ecological investigation and/or forest inventory is the most important determinant of subsequent sample protocols, estimation procedures, and data management efforts. Afore mentioned delineations among DDW components is specific to the US national inventory but roughly align with international commonalities^22^. Coarse woody debris often has a minimum diameter and length of 10 cm and 1 m, respectively^22^. Such minimum diameters of CWD are often adjusted to match classifications of FWD, as was the case in the US where the CWD minimum diameter was adjusted downward to align with FWD fuel classification systems. Regardless of Nation and/or forest type, the classification of DDW decay remains a qualitative but very necessary variable. Almost all inventories of DDW have some indicator of stage of dead wood decay whether a binary solid versus rotten classification or a five stage decay class system^22^. For those developing their own DDW inventories it is suggested that they consult local inventory techniques in the context of national/international standards^20^ and the goals of their resource inventory (e.g., wildlife habitat or fuel loadings).

### Plot Measurement Protocols, 2012-Present

**Figure 2:**
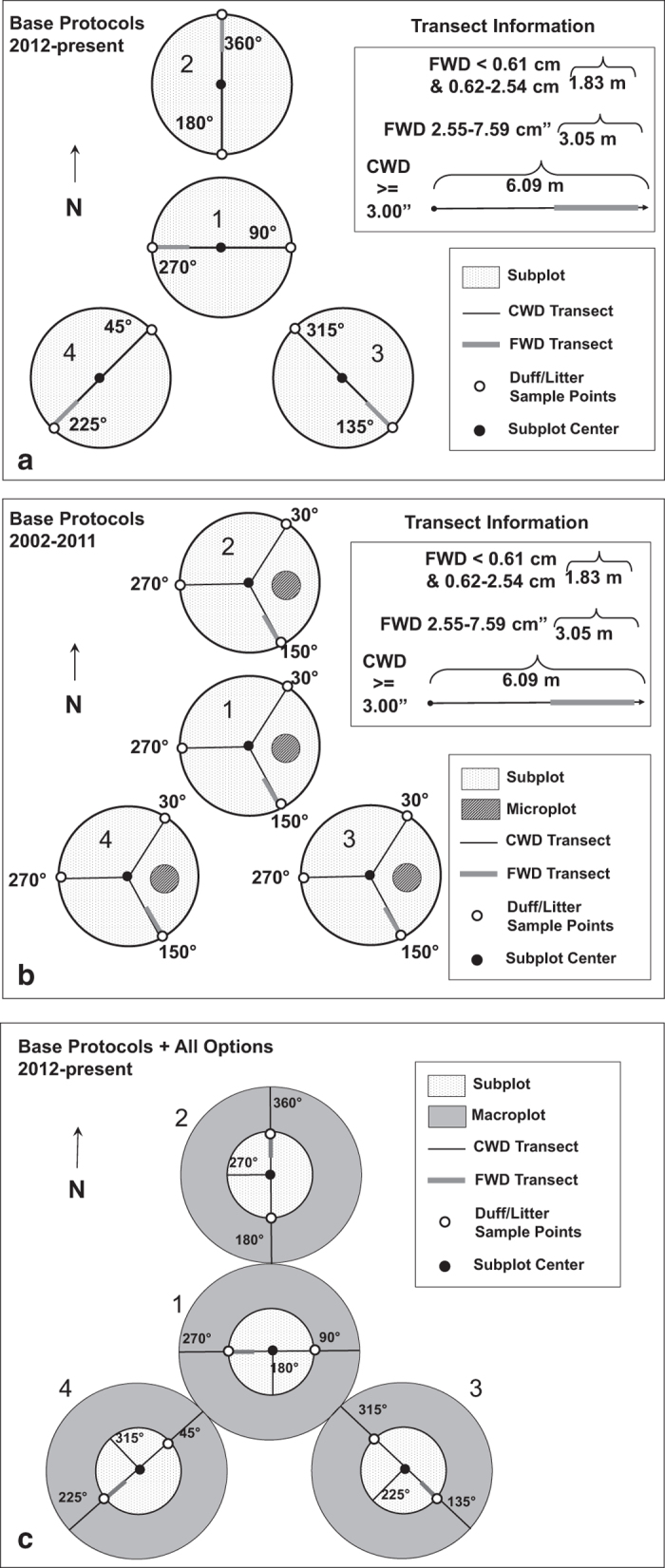
Illustrations of the downed dead wood plot measurement protocols for the most commonly employed protocols at the national scale, 2002 to present. (**a**) National base 2012 to present. (**b**) National base 2002 to 2011. (**c**) National base plus all options 2012 to present.

Dead wood information is collected in imperial units on FIA field plots (known as Phase 2 plots) with varying sample intensities across the country (from all Phase 2 plots [base intensity] to a subset of 1/16^th^ of Phase 2 plots). The design is based in a spatially balanced sample^37^ of one plot every ca. 24 km^2^. Each inventory plot consists of four points arranged in a cluster with one point at the center and three points oriented from the central subplot at 0, 120, and 240 degrees (Fig. 2). Each of these four points are referred to as subplots. The distance from the center of the central point to the center of the surrounding points is 36.58 m. These four points form the center of fixed-area plots used to tally live and standing dead trees, the origin of transects used to tally CWD pieces and FWD, and the center of point clusters used to estimate duff/litter biomass.

Because the FIA plots are located at random, the plot footprint may straddle areas with very different characteristics. If this is the case, the plot footprint is mapped into separate condition classes, and the population parameters (e.g., standing trees, downed logs) are assigned to the condition class in which they are located. The first condition class defines whether the area is forested, not forested, covered with water, or not accessible for measurement. Only the forested portion of the plot is measured. The forested area could be further divided depending on ownership, whether it is in a Congressionally-designated reserve, forest type, stand size class, regeneration status and tree density^38^.

Large pieces of downed wood that meet the criteria described in the previous section are tallied if a transect intersects the centerline of the piece. There are two, 7.32 m transects per subplot, arranged in opposing orientations (Fig. 2). If a transect straddles two or more condition classes, the transect is segmented, and the distance from the plot center to the condition class boundary is recorded. If a piece is intersected, the species, diameter at the point of intersection, degree of decay (1–5 scale from less decayed to more decayed) and condition class where it falls are recorded. If the piece is hollow, the diameter of the hollow section at the intersection point is also recorded. Additional measurements may be taken, depending on the region, including the inclination of the piece at the point of intersection.

In the rare event that that the transect intersects a large accumulation of wood (residue pile) that prevents measuring individual pieces separately, the length of the transect occupied by the pile, its compacted height (a visual estimate of the height of wood in the pile, excluding air, debris and pieces less than 7.62 cm in diameter), predominant species and predominant decay class are recorded. Residue piles are compact assemblages of CWD such that a distinct pile can be identified as separate from more typically scattered CWD. The pile is sufficiently distinct that measurements of its size and density of constituent CWD can be estimated. Pile sample protocols are necessary because some piles of CWD are so large that it is neither practical nor safe to attempt individual measurement of constituent, individual pieces of CWD using the standard LIS transect.

The number of fine woody debris pieces with transect diameter less than 0.61 cm and those with diameters 0.62 to 2.54 cm are tallied separately on a 1.83-m transect (4.27 m to 6.09 m along the CWD transect). The number of fine woody debris pieces with transect diameter of 2.55 to 7.59 cm are tallied on a 3.05-m transect (4.27 m to 7.32 m along the CWD transect). The depths of duff and litter layers are measured separately at the end of each of the two CWD transects on each subplot.

For further detailed plot measurement information please refer to the field guides for collection of both the standard forest inventory plots^39^ and DDW inventories^34^.

### Plot Measurement Protocols, 2002-2011

In 2012, the DDW inventory was overhauled based on user and field crew feedback within the context of QA/QC results and inventory analyses. At the national level and before 2012, CWD, FWD and duff/litter were only measured in the Phase 3 plots, which are a subset of the Phase 2 plots currently used. That sampling intensity was deemed too sparse to satisfy user needs to estimate DDW attributes by individual forest types and state across the US, thus the new protocols increased the number of plots sampled by a factor of 16. However, as this increase in sample intensity needed to be balanced with the cost of conducting the entire inventory, a number of DDW measurements were dropped in favor of a reduced set of measurements that could be affordably measured on the much larger set of plots. Efficiencies were gained through reducing transect length per plot, dropping some measurement variables to a subset that were highly desirable by user groups, and removing ecosystem components not directly associated with dead wood. Details of the pre-2012 protocols can be found a user guide^40^. The main changes in the national protocols were:

1. The number of transects per subplot was reduced from three to two, thus reducing the total transect length from 87.8 to 58.5 m per plot. The transect configuration also changed.
2. CWD piece measurements that occurred away from the transect intersection were eliminated, to minimize the time field crew spent walking the length of each CWD piece and the additional plot trampling. This includes the large- and small-end diameters and, more importantly, piece length. As a result, parameters such as total CWD volume, biomass, C, total length of CWD and CWD cover can still be estimated, but other parameters such as the number of pieces cannot.
3. Additional measurements intended to reduce some biases were introduced, including additional diameter measurements in hollow pieces and piece inclination.
4. The protocols to tally large accumulations of wood (piles) were completely overhauled. The current protocols integrate this measurement into the transect sampling protocols. Previously, piles were tallied using fixed-area plots^40^.
5. Transect lengths for FWD were changed from slope length (i.e., distance measured parallel to slope) to horizontal lengths, standardizing transect length and eliminating to need of a slope correction factor at the estimation phase.
6. Because the number of transects was reduced, so was the number of litter and duff points per plot (from 12 to 8).
7. Variables were added to clarify situations where there were obstructions to measurement such as early summer snowpack at some high elevation plot locations or seasonally flooded locations.

It should be emphasized that the current, reduced version of DDW plot measurement protocols has the flexibility to retain inventory components, such as increasing the DDW transect length and more detailed CWD measurements, if the resources and/or user support are present.

### Plot and population estimation procedures

The objective of an inventory such as FIA is to estimate parameters (e.g., CWD biomass or C per unit area) for a population (typically the State) or subpopulation (domain) of interest. For a forest inventory, a domain can be defined based on land classes (e.g., spruce/fir forests of Minnesota) or on attributes of the population element (e.g., logs of a particular species or decay class). Domain estimation is obtained by incorporating an indicator variable that selects population elements that meet the domain definition. The first step in the estimation procedure is to obtain an estimator of the parameter of interest in the domain of interest for each sampled plot. Once an estimator for each plot is calculated, population estimates are calculated from combined plots using the standard, FIA post-stratification estimators^40,41^. Because the only aspects of the estimation that require modification with the new protocols are the plot-level estimators, only those will be considered here with older and still valid population estimators still applicable^40^. Population estimators can be found in sections 3.2 and 3.3 of the older user guide^40^. It should be noted that these previous DDW estimators correct for plots that were partially missing (because portions of plot were hazardous or the owner denied access) using a correction factor ($\bar{p}$) for all the plots in each stratification category. For simplicity, we did not include these factors in the models below, but they can be found in section 3.1 of the older user guide^40^.

The estimation procedures of DDW plot and population attributes based on field measurement protocols used across the US from 2002 to 2011 were initially documented using imperial units^40^, based on the national FIA estimation procedures^41^. Given all prior published documentation is in imperial units, the estimators that follow will likewise use imperial units with conversion factors to metric provided (Table 1). The estimation fundamentals and theoretical justification documented in that publication are valid for the current design and will not be repeated here except when necessary to illustrate revisions to the estimation procedures due to changes to plot sampling protocols undertaken in the current DDW inventory (2012 onward).

1. Coarse woody debris estimator: This estimator has two components, one for individual pieces and one for piles, corresponding to the two summands in the equation below. In most situations, there are no piles intersected by the transect; therefore, the second component is 0. Written in a format aligned with the US DDW sampling protocols/precedents, the equation to estimate CWD volume for a single plot (which would substitute eqs 1 and 2 from Table 3.1 and section 3.1.1. of the older user guide^40^ becomes:

(1)ydi=1L∑j=1ns∑m=1ntr,s[C1Π28∑t[(DI)ijmt2−(DH)ijmt2]δijmtd+C2Π2∑t(PH)ijmt(PL)ijmtδijmtd]

Where:

*y*_*di*_ is the estimate of CWD volume per unit area in the domain of interest *d* for plot *i*.

*L* (ft) is the total length of CWD transect in the plot, according to protocol (typically 8*24 = 192 ft).

(*DI*)_*ijmt*_ (in) is the diameter at the transect intersection point of piece *t*, intersected by transect *m* of subplot *j* of plot *i*.

(*DH*)_*ijmt*_ (in) is the “diameter of hollow” at the intersection of piece *t*, intersected by transect *m* of subplot *j* of plot *i*.

*δ*_*ijmtd*_ is a domain indicator variable, which is 1 if the intersected piece or pile belongs to the domain of interest and 0 otherwise.

(*PH*)_*ijmt*_ (ft) is the compacted height of CWD at the intersection of pile *t*, intersected by transect *m* of subplot *j* of plot *i*.

(*PL*)_*ijmt*_ (ft) is the transect length at the intersection of pile *t*, intersected by transect *m* of subplot *j* of plot *i*. It is calculated as the “pile ending distance” minus “pile beginning distance”.

*n*_*s*_ is the number of subplots with transects (typically 4)

*n*_*tr*,*s*_ is the number transects in the *s* subplot (typically 2 per subplot)

*C*_1_ and *C*_2_ are constants to convert to proper units. In the database, the piece diameter in measured in inches, height and width of piles in feet, and transect length in feet. Then, *C*_1_=43560/144 and *C*_2_=43560provide estimates of the CWD volume per plot in ft^3^/acre. To obtain estimates in metric units, *C*_1_=21.1659 and *C*_2_=3047.89 provide estimates in m^3^/ha.

To estimate biomass and C, the measurements from every piece and pile are multiplied by its bulk density, which depends on the species and the degree of decay, and by a C conversion constant^42,43^. Thus equation 1 is modified to:

(2)ydi=1L∑j=1ns∑m=1ntr,s[C1π28∑t(DENS)ijmt[(DI)ijmt2−(DH)ijmt2]δijmtd+C2π2∑t(DENS)ijmt(PH)ijmt(PL)ijmtδijmtd]

Where:

*y*_*di*_ is the estimate of CWD biomass or C per unit area in the domain of interest *d* for plot *i*.

*L* (ft) is the total length of CWD transect in the plot, according to protocol (typically 8*24 = 192 ft).

(*DENS*)_*ijmt*_ (lb/ft^3^) is the bulk density or C density of piece *t*, intersected by transect *m* of subplot *j* of plot *i*, or of pile *t*, intersected by transect *m* of subplot *j* of plot *i*.

(*DI*)_*ijmt*_ (in) is the diameter at the transect intersection point of piece *t*, intersected by transect *m* of subplot *j* of plot *i*.

(*DH*)_*ijmt*_ (in) is the “diameter of hollow” at the intersection of piece *t*, intersected by transect *m* of subplot *j* of plot *i*.

*δ*_*ijmtd*_ is a domain indicator variable, which is 1 if the intersected piece or pile belongs to the domain of interest and 0 otherwise.

(*PH*)_*ijmt*_ (ft) is the compacted height of CWD at the intersection of pile *t*, intersected by transect *m* of subplot *j* of plot *i*.

(*PL*)_*ijmt*_ (ft) is the transect length at the intersection of pile *t*, intersected by transect *m* of subplot *j* of plot *i*. It is calculated as the “pile ending distance” minus “pile beginning distance”.

*n*_*s*_ is the number of subplots with transects (typically 4)

*n*_*tr*,*s*_ is the number transects in the *s* subplot (typically 2 per subplot)

*C*_1_ and *C*_2_ are constants to convert to proper units. In the FIA database, the piece diameter is measured in inches, height and width of piles in feet, and transect length in feet. The provided bulk density and C density is measured in lb/ft^3^. Then, *C*_1_=0.15125 and *C*_2_=21.78 provide estimates of the CWD biomass or C per plot in short tons/acre (a short ton = 2000 lbs). To obtain estimates in metric units, *C*_1_=0.33905 and *C*_2_=48.8233 provide estimates in Mg/ha.

As the cover and total length of CWD across a forest stand is critical to numerous wildlife habitat assessments^44^ we provide these additional estimators with the cover of CWD per unit area:

(3)ydi=100π2L∑j=1ns∑m=1ntr,s[∑t(DI)ijmt12δijmtd+∑t(PL)ijmtδijmtd]

Where:

*y*_*di*_ is the estimated percent cover of CWD in the domain of interest *d* for plot *i*.

To estimate the total length of CWD pieces per unit area:

(4)ydi=Cπ2L∑j=1ns∑m=1ntr,s∑tδijmtd

Where:

*y*_*di*_ is the estimated linear length per unit area of CWD pieces in the domain of interest *d* for plot *i*.

*C* is a constant to convert to proper units. In the database, piece length is measured in feet. Then, *C*=43560 provides estimates of the linear length of CWD in ft/acre. To obtain estimates in metric units, *C*=32808.4 provides estimates in m/ha.

2. Fine woody debris estimator: This FWD estimator deviates slightly from the 2008 procedures^41^, as the new protocol uses horizontal transect length, rather than slope length. The new estimator, for each FWD size class, is:

(5)ydi=Cπ28L∑j=1ns∑k=1Kijnijk(QMDI)ijk2δijkd

Where:

*y*_*di*_ is the estimate of FWD volume per unit area, for each size class, in the domain of interest *d* for plot *i*.

*L* (ft) is the total length of FWD transect in the plot, according to protocol (typically, 24 ft for the 0–0.24 and 0.25–0.9 in diameter classes, and 40 for the 1.0–2.9 in diameter class)

*n*_*ijk*_ is the number of FWD pieces in the diameter class of interest in condition class *k* of subplot *j* of plot *i*.

${\left(QMDI\right)}_{ijk}^{2}$ (in) is the quadratic mean diameter (i.e., diameter of a piece with average cross-sectional area) of the diameter class of interest in condition class *k* of subplot *j* of plot *i*. The quadratic mean diameters can be taken from a FWD diameter study^45^.

*δ*_*ijkd*_ is a domain indicator variable, which is 1 if the condition class belongs to the domain of interest and 0 otherwise.

*n*_*s*_ is the number of subplots with FWD transects (typically 4)

*K*_*ij*_ is the number of condition classes in subplot *j* of plot *i*.

*C* is a constant to convert to proper units. In the database, diameter classes and QMDs are measured in inches and transect length in feet. Then, *C*=43560/144 provides estimates of the FWD per plot in ft^3^/acre. To obtain estimates in metric units, *C*=21.1659 provides estimates in m^3^/ha.

Because individual species and decay classes are not recorded for FWD, to estimate the biomass or C, the estimated volume is multiplied by a bulk density or C density constant according to forest type^42,43^.

3. Duff and litter: The depth of the duff and litter are currently estimated in 8 points per plot, instead of 12 (prior to 2012). For each attribute, the volume (ft^3^/acre) of duff or litter in the domain of interest *d* for plot *i* assigned to stratum *h*, is:

(6)ydi=C∑j=1ns∑m=1np,syijmδijmdnsnp,s

Where:

*y*_*ijm*_ (in) is the depth of the duff or litter at the end of transect *m* of subplot *j* of plot *i*.

*δ*_*ijmd*_ domain indicator variable, which is 1 if the point at the end of transect *m* of subplot *j* of plot *i* assigned to stratum *h* belongs to the domain of interest *d* and 0 otherwise.

*n*_*p*,*s*_ is the number points in the *s* subplot (typically 2 per subplot)

*C* is a constant to convert to proper units. In the database, litter and duff depth are measured in inches. Then, *C*=43560/12 provides estimates of litter or duff volume per plot in ft^3^/acre. To obtain estimates in metric units, *C*=253.991 provides estimates in m^3^/ha.

To estimate the biomass or C in the litter and duff layers, the estimated volume is multiplied by a bulk density or C density constant according to forest type (Table 1).

### Code availability

All DDW data and associated ancillary information (tree and plot level information) is managed in an Oracle database on US government servers. A brief user guide^46^, reference table^47^, and all the PL-SQL code^48–53^ used in processing can be found in a digital repository. All inventory data is publicly available on accessible servers in various formats (xls, csv, etc) in the FIA Datamart (Data Citation 1) and via a digital repository (Data Citation 2).

## Data Records

The data provided in the Dryad DDW database contain all national core DDW available at the time of publication. As the DDW inventory is a component of the US National Forest Inventory, there are defined requirements for vetting data included in the national database along with a “corporate” data structure. Not only are all data management procedures documented, but final data must be made publicly available except in situations where personal information would be revealed (e.g., exact plot coordinates and the identity of private forest owners).

The data management process is initiated on each inventory plot where data recorders are loaded with a data collection program developed specifically for the DDW inventory. The data collection program guides field crews through the DDW data collection process and checks measurements based on the decades of data from DDW inventories. For example, the size distribution of CWD is well known for the US such that CWD diameter measurements larger than the 99^th^ percentile would be flagged in the data collection program for verification by the field forester. The field data are then processed by a data management team that conducts additional edit checks (for measurement errors and logic) while also conducting additional computations to enable population estimation (e.g., total CWD by state). Next, a forest resource analyst examines the data and associated population estimates of DDW resources. Once the DDW data pass this final verification, they are loaded into the publicly available database (Data Citation 1). The DDW data are organized in a number of data tables that can be roughly delineated as plot/site and DDW sampling information, DDW variable measurement data, and reference information. The FIA database is relational in that all tables can be linked via “control numbers” for individual plots across space and time.

Although a large number of data tables comprise the FIA database, only a few are necessary for DDW estimation/analysis, namely the plot, condition, and transect tables. Additional population estimation tables contain details regarding estimation units, post-stratification information (e.g., stratum weights), and assignment of individual plots to said estimation processes. Details regarding each table and field are publicly available both at the online FIA library^54^ and at digital repositories^55,56^. What follows below is a general outline of data tables of most relevance to using DDW data, although additional tables can be brought to bear, such as the tree seedling table for more comprehensive forest ecosystem analyses.

### Plot, condition, and visit data

The plot table contains information on approximate plot location (spatially perturbed due to federal privacy laws), measurement dates, and survey information relevant to conducting an inventory across the US (e.g., cycle of the survey and field manual version used on the plot). The condition table contains much of the non-tree measurement information typically measured on a forest inventory plot, such as whether the condition is forested or not and, if so, ownership information, forest type, stand age, disturbances, and forest management activities. There can be more than one condition per plot owing to delineations between ownerships and/or forest types across the spatial extent of a plot (i.e., changing forest conditions across the plot footprint). Perhaps the most important field in the condition table is condition identification (CONDID) which identifies each unique condition on an individual plot. Specific sampling locations on a plot depend on the plot and condition identifiers. The DDW visit table contains information regarding which set of plot sample protocols were used on any particular inventory plot at any point in time. An important field in this table is the sample kind code (SMPKNDCD) that identifies which plot protocols were used. Another important field is the quality assurance status code (QASTATCD) that denotes whether the data record represents a standard inventory plot or QA data (i.e., blind remeasurement for the purpose of statistical control of the national DDW sample). Finally, the transect segment table provides the total length of each sampled transect in each condition, which is needed for calculation of DDW plot and population estimates.

### Downed dead wood field data

Data tables for the actual measurements of DDW attributes are allocated to five data tables: coarse woody debris, fine woody debris, duff/litter/fuel, microplot fuel, and residue pile. The microplot fuel table contains data collected on the heights and coverage of shrubs and herbs prior to 2012, but these measurements are not currently taken. Data rows in the coarse woody debris table relate to individually unique pieces of CWD sampled along the transects on subplots/plots. This table includes all measured attribute information for each CWD piece (e.g., transect diameter and species), along with sampling related information such as transect location and distance from subplot center. In contrast, data rows in the FWD table are unique for each sampled FWD transect on each subplot for each unique condition. As such, total counts of FWD by size class are included in this table. Data rows in the duff/litter/fuel table are unique to each sample location point on each subplot transect, providing estimates of duff and litter depth at these sample points.

### DDW estimates and reference data

Estimates of DDW attributes (e.g., CWD C or biomass) are provided at the plot/condition level (i.e., not expanded to domains of interest) in the DDW calculation table (DWM_cond_calc). Reference information (decay reduction factors by CWD species and decay class) for the purposes of estimating individual DDW attributes at the plot or population level are included in the tree species reference table.

## Technical Validation

A portion (2–5%) of DDW field plots are revisited annually by field crew supervisors/trainers who conduct a second DDW measurement independent of the first (i.e., blind remeasurement). The resulting blind datasets are used to both evaluate data quality by field crew members and maintain statistical control of the sample. To achieve an acceptable level of repeatability, minimum quality standards are established for every DDW measurement variable (e.g., ± one decay class for the CWD decay class assessment) (see Table 1 in quality control analysis^57^). In addition to annual monitoring of field crew and maintenance of data quality, the quality control results are published periodically^57^. These analyses help guide decisions about sampling and determine where best to allocate resources. For example, due to poor repeatability, shrub/grass/herb measurements were removed from the DDW plot protocols starting nationally in 2012. Repeatability for FWD was also poor; however, given its importance in fire behavior models, the measurements were retained in the post-2012 field protocols.

In addition to the various sources of measurement error^57^, other factors contribute to uncertainty in estimates of DDW. Ultimately, most users are interested in DDW values at the plot and population level. Additional sources of error are introduced in those calculations, such as stratum weights, which can increase uncertainty. Similarly, assumptions associated with estimates of bulk density and C content may also influence population estimates^58^. While various sources of error have not yet been propagated for DDW across broad regions, inclusion of estimates of uncertainty in the future will provide confidence in the values and improve interpretation of the data.

## Usage Notes

As the DDW data are freely available for download in several formats from the FIA Datamart there are various routes users can take towards managing and using the data. As previously mentioned, the DDW condition calculation table contains estimates of DDW attributes of most interest to users. However, all field measurements associated with the condition/plot level DDW estimates may be of interest to users. For example, the CWD table has valuable information on the species composition and size distribution of CWD pieces across the US forests. The FIA online library contains the field guides from every year of the DDW inventory (although since 2012 there have been no changes to the DDW plot protocols) in addition to the detailed database user guides. As the DDW inventory is an annual inventory, new data are posted by state at least once a year. The major issues that DDW data users should be aware of pertain to actual plot locations, comparisons between DDW plot protocols, and regional differences.

The FIA database that contains DDW data are only legally allowed to provide perturbed plot locations (shifted in latitude and longitude) to protect the privacy of private land owners. The actual locations are typically perturbed by less than 1 km, allowing coarse alignment with spatial datasets of similar resolution (e.g., 4 km gridded climate data). If users require actual plot locations for use with finer resolution spatial products, such as LANDSAT imagery, the Spatial Data Services Center of FIA must be contacted for options whereby a user may gain access to the coordinates.

As the national core of DDW plot protocols (described herein) have changed over time, effectively they should not be treated as repeated measures at the condition/plot level, as doing so may produce spurious results. Although the same inventory plot may have been measured for DDW in 2010 and 2015, shifted transects locations and lengths likely result in differences of DDW estimates attributed as much to protocol changes as to true differences. That being said, a large number of DDW plots were remeasured from 2002–2012 using the same protocols, and likewise DDW plots measured since 2012 are currently being remeasured with the same protocols. The difference between the 2002–2011 and 2012-present DDW inventory protocols represent the largest discontinuity of measurement protocols that users should be aware of.

Finally, although the basic DDW plot protocols consistently applied across the US have been described herein, the opportunity exists for states/regions to add additional variables or lengthen sample transects. Due to this program feature, users should pay close attention to the sample kind code in the DDW visit table, as it describes which set of plot measurement protocols were employed.

## Additional information

**How to cite this article**: Woodall, C. W. *et al*. The downed and dead wood inventory of forests in the United States. *Sci. Data*. 6:180303 doi: 10.1038/sdata.2018.303 (2019).

**Publisher’s note**: Springer Nature remains neutral with regard to jurisdictional claims in published maps and institutional affiliations.

## Supplementary Material



## Figures and Tables

**Table 1:** Downed dead wood database tables, commonly used fields, and usage notes.

Table	Description	Commonly Used Fields (column name)	Usage Notes
Plot	Location and site information	Control Number (CN)	This table can be linked to all the DDW tables via the CN field which is referred to as the plot control number (PLT_CN) in all DDW tables
		Latitude (LAT)	
		Longitude (LON)	
		Measurement Year (MEASYEAR)	
Condition	Forest attributes	Forest Type (FORTYPCD)	Contains a limited set of summary information on ownership and forest attributes
		Stand Age (STDAGE)	
		Live tree basal area (BALIVE)	
		Ownership class (OWNCD)	
		Disturbance codes (DSTRBCD)	
DWM visit	Describes DDW sampling information	Inventory Year (INVYR)	Critical information for identifying whether a plot was a standard forest inventory plot or a quality control plot. In addition, the specific type of sample protocols are identified.
		Quality assurance status code (QASTATCD)	
		Sample kind code (SMPKNDCD)	
		Coarse woody debris sample method (CWD_SAMPLE_METHOD)	
		DWM sampling status code (DWM_SAMPLE_STATUS_CD)	
		DWM number of subplots (DWM_NBR_SUBP)	
DWM coarse woody debris	Detailed information for individual pieces of CWD sampling on each plot	Subplot number (SUBP)	There are numerous calculated attributes for each CWD piece measured along each transect in this table, please consult full database documentation for details
		Transect number (TRANSECT)	
		CWD piece number (CWDID)	
		Species code (SPCD)	
		Decay class code (DECAYCD)	
		Transect diameter (TRANSDIA)	
		Dry biomass of piece (DTYBIO_AC_PLOT)	
DWM duff, litter, fuel	Plot-level measurements of duff and litter	Sample location code (SMPLOCCD)	Due to seasonal obstructions to measurement such as snow or flooding the measurements may be null…refer to non-sampled reason codes
		Duff depth (DUFFDEP)	
		Litter depth (LITTDEP)	
		Fuelbed depth (FUELDEP)	
		Duff, litter, fueldbed sample method (DLF_SAMPLE_METHOD)	
DWM fine woody debris	Measurement information for fine woody debris counts on plots	Small-size class count (SMALLCT)	Although there are numerous fields in this table, the counts of FWD By size are the most important
		Medium-size class count (MEDIUMCT)	
		Large-size class count (LARGECT)	
DWM residual pile	Measurement information for residue (harvest) piles identified on a plot	Compacted height (COMP_HT)	This table contains substantial measurement information from pre-2012 sample protocols, the transect sampling of piles are additional fields in this table.
		Decay class code (DECAYCD)	
		Pile beginning horizontal distance (HORIZ_BEGNDST)	
		Pile ending horizontal distance (HORIZ_ENDDIST)	
		Pile sample method (PILE_SAMPLE_METHOD)	
		Species code (SPCD)	
		Transect azimuth (TRANSECT)	
DWM transect segment	Sampling information for transects on each plot	Transect code (TRANSECT)	Typically this information is only used when calculating DDW attributes at the condition, plot, and population level as it identified which parts of transects on subplots/plots were sampled (i.e., part of the domain of interest)
		Segment number (SEGMNT)	
		Condition identification (CONDID)	
		Horizontal length (HORIZ_LENGTH_	
		Beginning horizontal distance (HORIZ_BEGNDIST)	
		Ending horizontal distance (HORIZ_ENDDIST)	
		Segment status code (SEGMNT_STATUS_CD)	
Condition DWM calculation	Plot-level estimates of DWM attributes	All volume, biomass, and carbon estimates by DWM attribute for the condition, unadjusted at plot level, and adjusted at plot level	This table is the most widely used by analysts as the calculation of numerous DDW attributes at the condition/plot level have already been conducted. Connecting this table to plot and tree level tables enables a host of analyses.
Forest type group reference	Estimation constants by forest type group needed for producing plot and population estimates of duff, litter, FWD, and piles of CWD	Density (e.g., DUFF_DENSITY) Carbon content (e.g., DUFF_CARBON_RATIO) Decay ratio (e.g., FWD_DECAY_RATIO) FWD diameter (e.g., FWD_SMALL_QMD)	Density estimates are provided in bulk density units (lb/ft^3^) while carbon content and decay reduction factors are in terms of ratios, FWD diameters are provided in inches.
Species reference	Estimation constants by tree species type group needed for producing plot and population estimates of CWD	CWD specific gravity (WOOD_SPGR_GREENVOL_DRYWT) CWD decay ratio (e.g., CWD_DECAY_RATIO1) CWD carbon content (DWM_CARBON_RATIO)	The density of CWD is provided in terms of specific gravity (unitless) while CWD decay ratios are provided for the individual decay classes one through five.
